# Evolving through multiple, co-existing pressures to change: a case study of self-organization in primary care during the COVID-19 pandemic in Canada

**DOI:** 10.1186/s12875-024-02520-3

**Published:** 2024-08-05

**Authors:** Patricia Thille, Anastasia Tobin, Jenna M. Evans, Alan Katz, Grant M Russell

**Affiliations:** 1 Department of Physical Therapy, College of Rehabilitation Sciences, University of Manitoba, R106-771 McDermot Ave, Winnipeg, MB R3E 0T6 Canada; 2 DeGroote School of Business, McMaster University, 1280 Main Street West, L8S 4L8 Hamilton, ON Canada; 3 Manitoba Centre for Health Policy & Departments of Community Health Sciences and Family Medicine, University of Manitoba, 408-727 McDermot Ave, Winnipeg, MB R3E 3P5 Canada; 4 Department of General Practice, Monash University, 553 St Kilda Road, Melbourne, VIC 3004 Australia

**Keywords:** Primary care, Case study, Self-organization, Actor-network theory, Organizational routines, COVID-19 pandemic

## Abstract

**Background:**

Primary care is often described as slow to change. But conceptualized through complexity theory, primary care is continually changing in unpredictable, non-linear ways through self-organization processes. Self-organization has proven hard to study directly. We aimed to develop a methodology to study self-organization and describe how a primary care clinic self-organizes over time.

**Methodology:**

We completed a virtual case study of an urban primary care clinic from May-Nov 2021, applying methodological insights from actor-network theory to examine the complexity theory concept of self-organization. We chose to focus our attention on self-organization activities that alter organizational routines. Data included fieldnotes of observed team meetings, document collection, interviews with clinic members, and notes from brief weekly discussions to detect actions to change clinical and administrative routines. Adapting schema analysis, we described changes to different organizational routines chronologically, then explored intersecting changes. We sought feedback on results from the participating clinic.

**Findings:**

Re-establishing equilibrium remained challenging well into the COVID-19 pandemic. The primary care clinic continued to self-organize in response to changing health policies, unintended consequences of earlier adaptations, staff changes, and clinical care initiatives. Physical space, technologies, external and internal policies, guidelines, and clinic members all influenced self-organization. Changing one created ripple effects, sometimes generating new, unanticipated problems. Member checking confirmed we captured most of the changes to organizational routines during the case study period.

**Conclusions:**

Through insights from actor-network theory, applied to studying actions taken that alter organizational routines, it is possible to operationalize the theoretical construct of self-organization. Our methodology illuminates the primary care clinic as a continually changing entity with co-existing and intersecting processes of self-organization in response to varied change pressures.

**Supplementary Information:**

The online version contains supplementary material available at 10.1186/s12875-024-02520-3.

## Background

Primary care is often described as slow or difficult to change, [1–3] as are health systems more generally [4, 5]. Yet, this argument often relies upon particular assumptions about change – that change is controllable, predictable, and/or the result of linear planned approaches. Many argue that ‘planned change’ approaches, which assume controllability and predictable outcomes are possible, mislead and can cause failure [6–9]. 

When health care organizations are observed closely, small alterations in relationships can create big, unexpected impacts, while in other situations, big redesigns seem to have minimal impact [9–11]. Complexity theorists suggest this unpredictability is the result of ongoing influence of varying initial conditions, values, attractors, relationships, communication, resources and more, all of which shape how each primary care clinic changes over time [7, 12, 13]. As a result, both purposefully-initiated changes and emergent ones are unpredictable in terms of direction and outcomes [7]. 

Many argue the field of implementation science needs new guiding theories and methods to study the dynamic changes and relationships within primary care organizations, proposing complexity theory as a better foundation for the study of change [7–9, 12]. Starting from the assumption that primary care clinics exhibit complexity dynamics, [6–8] this means that clinics change through a process called *self-organization*, the results of which are unpredictable and non-linear. Proponents of a complexity lens encourage more non-experimental and mixed-methods approaches to study dynamic relationships involved in practice change [9, 10, 14]. 

There are methodological challenges in studying dynamic relationships and self-organization processes within organizations over time. In part, this is because primary care is subject to many co-existing attempts to alter services and/or structures. Each clinic continually navigates a confluence of change agents^1^ and change pressures, both internal and external [15]. In many Canadian provinces, for example, governments have supported the establishment of primary care interprofessional teams and new models of care in some settings [16, 17]. This external/governmental drive results in new professions joining primary care. Each new health professional joining a primary care clinic becomes an internal change agent, doing work to ‘fit’ within the clinic and its services [18]. Concurrently, other agents may try to influence primary care services, including: disease-based organizations (e.g. Heart & Stroke Foundation); government-funded organizations (e.g. Canadian Task Force on Preventive Health Care); university researchers; and private corporations (pharmaceutical and IT companies). Each agent seeks particular changes and outcomes, which may conflict with others. Changes made may create new problems for the clinic to manage. Finally, as disasters such as pandemics remind us, extreme events also necessitate change to align primary care services with new policies, resource availability, and community health needs.

Much practice change research focuses on successful implementation of a particular change desired by a specific group of actors. Even when the research is high quality, this approach limits our understanding of how primary care clinics navigate change. As a result, many methodological approaches and empirical explorations fall short of producing contextually-specific depictions of how self-organization occurs [3, 9]. In addition, explicit or implicit theories have led some researchers to take up more reductionistic approaches that list presence or absence of certain types of factors, labeled as facilitators or barriers [3]. In contrast, complexity-oriented scholars argue that we need to let go of additive and linear models of change, [19, 20] which can oversimplify reality. In primary care, oversimplifications can result in poorly conceived implementation approaches [3, 9].

In response, our complexity-theory informed project bridges methodological tenets from actor-network theory to prospectively study change in a Canadian primary care practice over a six-month period. Actor-network theory emphasizes studying action. We chose to focus analytic attention on the actions and processes of changing organizational routines. While our intent is to make a methodological contribution to the study of self-organization, we hope readers also find this a meaningful descriptive case study of ongoing disruption to clinical and administrative routines during the COVID-19 pandemic.

### Theoretical foundations

Complexity theory focuses on how systems work. Complexity theory starts from the assumption that what is made real – such as the everyday activities in a primary care clinic – is the result of humans and non-humans (e.g., clinicians, electronic medical record (EMR), physical clinic spaces) coming into relationships of mutual interdependence [19, 21]. Facing pressures from within or from external environmental influences (e.g., health policies; community context), agents within will adjust routines and relationships to adapt in a way that better fits their current situation, [10, 22] though the particular adjustments they make are unpredictable [6]. Any stability in particular routines (or patterns, as per Anzola and colleagues [23]) is a temporary accomplishment [19] and never pre-determined; instead, temporary forms of “order comes from the actions of interdependent agents who exchange information, take actions, and continuously adapt to feedback about others’ actions rather than from the imposition of an overall plan by a central authority” [24]. ^p343^ With no central control over system-wide outcomes, agents act within their local environment, shaping and being shaped by the “complex responsive processes of relating” [7]. ^p1^ No agent is static or consistently predictable; instead, “each agent is continually acting and reacting to what other agents are doing”, [25]^p31^ which is called co-evolution. As a result, change is non-linear “and rarely explained by simple cause–effect relationships”. [24]^p342−3^ Change attempts can always fail, which in the most extreme cases can result in collapse [12]. 

When applying complexity theory, we assumed primary care practices have many change pressures, both internal and external, and that change occurs in unpredictable ways via *self-organization*. No singular definition of self-organization exists [23]. We chose the following, after considering others: “the way in which agents interact to coordinate their own circumstances, workplaces, processes and procedures, such that they order their work and they autonomously, or semi-autonomously, organize their localized behavior”. [10]^p6 ^The emphasis is on the dynamics of action, recognizing interdependencies both within and with external influences. The actions taken may result in a re-ordering of activities within the organization, without assuming long-term stability.

The methodological challenge we sought to address was to make self-organization processes observable. To do so, we applied tenets of actor-network theory (ANT), which is not a ‘theory’ per se, but a methodological toolkit developed within science and technology studies based on a relational ontology that considers both human and non-human actors. In ANT, reality does not precede routine practices, but is instead shaped by them [26, 27]. ANT studies approach “everything in the social and natural worlds as a continuously generated effect of the webs of relations within which they are located. It assumes that nothing has reality or form outside the enactment of those relations.” [28]^p.141^ ANT studies are always situated, local, describing action in a time and place as it unfolds through relations – including both relations that are attempted but fail, and those which are established [27, 29]. ANT has strong foundations in sociology with ethnomethodological roots, [30] though exceeds any one discipline.

Unlike the approach used by some primary care scholars applying complexity theory, ANT is not concerned per se with ‘systems’ or ‘organizations’ or their conceptualization. However, based on decades of ethnographic studies, ANT shares many core assumptions about action and change with complexity theory. Like complexity theory, ANT does not shy away from or attempt to simplify ‘messiness’; in contrast, it is explicitly anti-reductionist, approaching reality as emergent and continually changing in unpredictable ways from a generative flux of relations among human and non-human actors [29]. Actor-networks that become known through research are limited moments in the flux [29]. 

Similarly to complexity theory, ANT understands stability over time as unusual; decay, change and creation are more common [27]. If a stable order of action occurs for a time, it is because some mechanism exists that stabilizes it within the actor-network producing the action [27]. An actor never acts alone and can be made to act by others, which the hyphenated term of ‘actor-network’ signifies [27]. Acting requires the capacity to act, which depends upon relations to others. Responsibility is distributed into a dispersed network of interdependencies and co-responsibilities, [31] rather than centrally controlled. Attempts to change actor-networks acting together involves *translation*, which “involves creating convergences … by relating things that were previously different.” [32]^p211^ Translations can always fail, thus attempts at change have no foregone conclusion; there are always contingencies that create many possible outcomes [32]. 

Generated from ethnographic studies of science, [30] health care, [26] ecological interventions, [33] and other contexts, ANT orients researchers to stay close to the trail of action, observing the actor-networks involved and what is brought into being, however temporarily [27]. By closely following the trail of action, it is possible to explore how changes within actor-networks can intersect and interfere with each other, when change stops, and when one change cascades into others [27]. 

## Methodology and methods

### Case study

Complexity theory studies of primary care have often used case study as a methodology. Case studies are studies of the particular, “a way to unravel the complexity of one demarcated entity” chosen by the researcher, who seeks to understand a naturally occurring phenomenon in the setting [34]. ^p1150^ Like organizations, [23] a case has boundaries – albeit fuzzy ones – with working parts within that often have a purposive aspect to them [35]. Researchers choose the case and enact the context for a case study, [36] and can search for links to entities external to but influential on the chosen case, as well as historical information about the setting that influences the present [34]. These assertions parallel how complexity theory understands ‘systems’ or organizations, making case study an appropriate methodology for our project. Our case study aimed to make both a methodological contribution to the study of self-organization and offer a meaningful descriptive case study of a Canadian, urban primary care clinic navigating multiple pressures to change, during May – Nov 2021, the second year of the COVID-19 pandemic.

In case studies, the primary focus is what is happening within the researcher-selected boundary [37]. With a goal of exploring processes of self-organization within a primary care clinic, to bound and focus our case, we decided to study actions taken to alter organizational routines, defined as “a repetitive, recognizable pattern of interdependent actions, involving multiple actors”. [38]^p96^ These recognizable patterns exist through repetition and recursion, but variation and new emergent routines are always possible through expressions of agency in situ [19, 38]. This is consistent with the theoretical understanding that any local order emerges from within, via relations [27, 39]. We chose to study changing routines because we anticipated self-organization would be well-illustrated by this focus.

Case studies require researchers to identify the focus of the case among many possibilities, such as a person, an organization, a process, an event, or a system [35]. We aimed to make visible a naturally occurring process theorized in complexity theory: self-organization within a primary care clinic. In Stake’s terminology [35], this focus makes ours an instrumental case study. Instrumental case studies examine the case primarily to gain insight into another issue; that is, the case supports better understanding of a different entity that the researcher chooses. Despite the instrumental aim, such case studies still demand a deep understanding of the case to pursue the broader interest.

The power of case studies is in the depth of the study of the particular, so choosing a ‘good’ case is important. Purposive sampling is typical, aiming for ‘learning potential’ rather than representativeness [34]. Our goal was to test and refine methods to prospectively study self-organization. To stress test our methods, we recruited a primary care clinic with known, immediate relationships with other co-located teams; while working closely together, these co-located teams have different governance and performance expectations. We delineated the boundaries of our case as the primary care clinic as the ‘internal’, in terms of the group working together under that one governance structure, while anticipating other ‘external’ co-located team members and more distant external bodies may become involved in changes we observed.

### Recruitment and ethics

The project was approved by both the University of Manitoba’s health research ethics board, and a health system review body. The study was carried out in accordance with relevant guidelines and regulations outlined in the ‘Ethics approval and consent to participate’ disclosure section.

Recruitment was a three-step process. We first proposed the study to a clinic’s co-leaders, then presented our study at a team meeting. We sought written confirmation of interest from the individual team members via email, avoiding perceived coercion by clinic leaders. Once interest was confirmed, we completed individual written consent processes separately for observation of team meetings and interviews. Each participant selected their own pseudonym; in the rare case where they did not, we assigned one. We use initials of their pseudonyms in the manuscript.

### Data collection

We created and collected multiple forms of qualitative data, including non-participant observation of meetings, document collection, key informant interviews, and weekly updates. First, a research team member (Tobin) was a non-participant observer in the pre-existing, virtual, biweekly 1.25 h meetings with the full team for a six-month period, May – Nov 2021. She wrote extensive field notes describing the discussions and decisions made in the meeting, with limited additions to describe the virtual scene [40]. Second, we collected documents related to these meetings, retrospectively and prospectively, from Jan 2020 through Nov 2021. Third, we interviewed key informants within the clinic – specifically, people we identified as closely involved in multiple change processes – with a semi-structured interview guide we created orienting to complexity theory concepts. Finally, after attempting a written method for weekly updates on change initiatives underway, we started weekly phone calls with an administrative leader in Sept 2021. Our case study had revealed this person to be a gatekeeper, or in ANT language, an ”obligatory passage point” [33]^p196^ for all organizational routine changes. She answered a series of structured questions on the progress of each of the different initiatives the clinic was undertaking. Appendix A contains both the interview and weekly update guides.

### Data analysis

#### Schema analysis

Our analytic process is guided by the actor-network theory emphasis on describing action, and our specific interest in describing the processes of changes to organizational routines over time. We adapted Rapport’s method of inductive schema analysis to develop triangulated descriptions of the actions that changed different organizational routines [41]. Schema analysis is a team-based approach where each member reviews a data text, creating what they deem to be an accurate summary including what they perceive as ‘essential elements’. Team members then compare summaries and co-develop a descriptive ‘meta-schema’ of the text, which is then used to support interpretation [41]. 

We adapted schema analysis in two ways. First, we created a chronological schema for each potential organizational change topic, rather than for each fieldnote, interview, or document which addressed multiple changes. Second, we explicitly and purposefully worked with theoretical concepts to help us identify what we thought were ‘essential elements’ rather than working inductively.

#### Our processes

Using NVivo 12, Tobin coded data to each of the different change-related topics raised in fieldnotes of team meetings, interviews, and weekly updates. She then arranged the data specific to each change-related topic in chronological order. Thille and Tobin then independently reviewed the chronological document, developing an individualized chronological summary or ‘schema’ of each particular change topic, focusing on how problems are identified and how different actors work in concert to attempt to create a new organizational routine. We reviewed and compared the pair of schemas for each change topic, flagging different interpretations, and returning to the original data to clarify, before finalizing a meta-schema for each change. The resulting meta-schema describes each change initiative, created with multiple data sources and analyst triangulation.

We then classified the different meta-schemas, confirming each was an active change initiative to clinical or administrative routines involving two or more members of the clinic. We found some topics were instead, (a) recent history (and thus valuable context), or (b) non-human actors in the ANT sense that shaped what was possible across multiple other changes (e.g. EMR, physical space), or (c) events that did not involve changing administrative or clinical routines (e.g. accreditation).

We held two member checking sessions to evaluate how well our methods illuminated the processes of self-organization: one with most members of the team (45 min), and one with the lead administrator of the clinic (60 min). After presenting, we solicited feedback. The team could respond in the meeting and/or via an anonymous survey, while the lead administrator responded verbally and reviewed our written summary of her feedback for accuracy.

Throughout the study, Thille and Tobin wrote analytic memos to enhance reflexivity, and track method-related challenges and decisions.

### Findings

In 2021, the clinic continued to self-organize in the face of changing health policies, unintended consequences of earlier adaptations, and quality improvement initiatives. In both our reconstruction of past changes made, and our observation of active changes underway to clinical and administrative routines, obvious non-human actors such as physical space, staffing, and technology affected self-organization, in addition to pandemic-related policy changes. Changing one actor (human or non-human) often created ripple effects, sometimes generating new problems. We first offer context about the clinic and the year prior to the prospective case study, then describe self-organization processes. We italicize phrases related to complexity theory within the narrative.

### North star clinic

The “North Star” primary care clinic is located in a mid-sized Canadian city. They had had stable leadership for over a decade, with a co-leadership structure of a Medical Director (W) and Team Director (C). At the time of study initiation, the primary care practice staff included 35 team members, including physicians, nurse practitioners, registered nurses, administrative staff and primary care assistants. The primary care clinicians have panels of patients, though also see patients beyond their panel (e.g., immunizations). North Star has governance connections with a larger regional body. The clinic is co-located with three other publicly-funded teams (not named to protect confidentiality), which each have their own governance. The clinic is a training site for medical students, family medicine residents, and other trainees.

North Star had several long-established methods to initiate and support change, including a method they called ‘process mapping’, an annual retreat, and resident-led quality improvement initiatives. North Star also held bi-weekly team meetings (1.25 h/meeting), as well as several more informal methods to share concerns, *make sense* of possible causes of the problem, and offer *feedback* on process changes being contemplated or underway in the clinic. For example, the office manager had ‘an open-door policy’, and a senior clerk had a daily habit of walking around the clinic, checking in with staff. In addition, different groups within the clinic had their own meetings.

### The first year of the pandemic

Unsurprisingly, key informants described the pandemic as causing *disequilibrium* in 2020, where existing clinical and administrative routines no longer fit the conditions. At initiation of the study (May 2021), the clinic was over a year into pandemic-related changes, including rapid uptake of virtual care, prescription renewal by fax, and the creation of both a COVID-19 vaccination clinic and a clinic for people ill with COVID-19, described briefly on Table 1. When asked about any periods of stability, two key informants laughed; the question was absurd in relation to their experience. As a clinic, they had created new routines in response to policy changes external to the clinic, the capacities and concerns of those working at North Star (e.g., safety), the physical and digital resources available to them, and the perceived needs of patients. For example, North Star and the other co-located teams had already adapted to the loss of the organization’s nurses due to periodic redeployments to help with the broader public health pandemic response. Redeployments changed resources and disrupted relationships, which could necessitate re-organization of various processes to keep primary care service delivery on track. Together, these varied change pressures created routines that were in place when we started the prospective study.

**Table 1:**
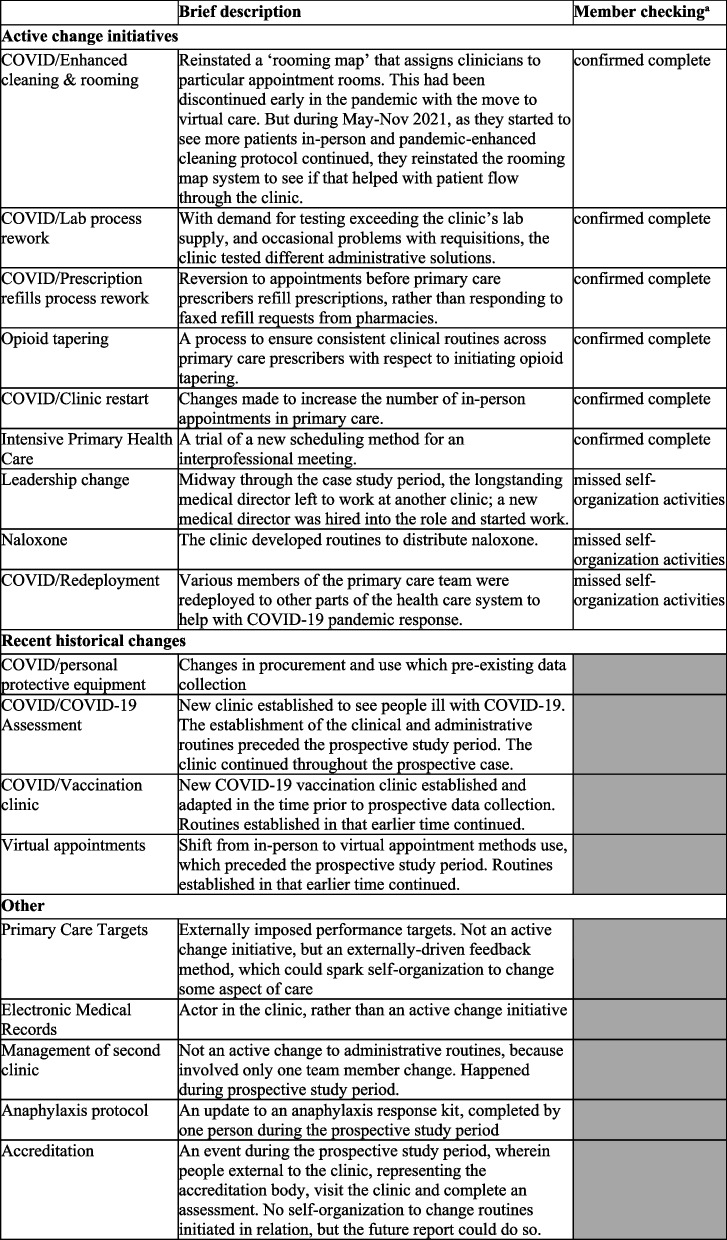
Change initiatives considered during the prospective case study period

^a^Member checking reflects feedback of participants to our study findings presentation; only active changes were presented, in full. Greyed-out boxes were not presented in full detail

### Observed self-organization affecting clinical and administrative routines

From the meta-schemas we wrote and via member checking, we identified nine active, co-existing ‘change initiatives’ to alter organizational routines. Each involved multiple people at the site working together to establish new or adapt existing clinical routines during the prospective study period. Member checking confirmed we described six of these well. For the three others, our methods missed some of details of the change processes, primarily because they were not discussed at full meetings. As mentioned above, we determined that four meta-schemas we wrote were part of the recent history of the clinic but were not subject to change during the prospective study period. The final set (“other”) were topics raised at meetings, but not themselves active changes. See Table 1 for a summary.

Some of these change initiatives are related, one affecting another, described in the narrative below.

### Tightly coupled routines: changing health policies sparking iterations of self-organization

At the team meeting on August 5th, 2021, JF (Medical Director) announced that the Region was “hoping” primary care clinics will shift their focus back to pre-pandemic clinical processes, anticipating COVID-19 restrictions would soon be lifted. North Star’s term for this was “clinic restart”. In addition, the Region expected the clinic to continue offering new services initiated during the pandemic – most notably, continuing to see non-rostered people with symptomatic COVID-19 referred to their COVID-19 assessment clinic. JF expected relaxation of physical distancing rules and increasing the ratio of inpatient-to-virtual appointments to 80/20. She explained this is, in part, due to concerns about delayed immunization and preventative screening – population-level consequences of the pandemic-related changes – as well as the Region’s push to increase patient intakes and panel sizes overall.

The team discussion of the clinic restart raised several closely related issues, highlighting *tightly coupled* clinical and administrative routines where a change in one process necessitates changes to others. First, greater numbers of in-person patient visits necessitated revision to the scheduling templates and providers’ availability on site. Second, team members agreed that in-person visits should be driven by patient preference, rather than those of health policy makers. As C commented: “The Region can say we would like you to do 80/20 but if we don’t have the uptake for that then that doesn’t make sense for us either. Locally we have to think about what our patients are asking for too”. Third, with more people visiting in person, JF flagged the need to ensure patients flow well through the clinic to avoid overcrowding in the waiting room. Similarly, MT (primary care assistant) added that they also need to avoid telephone “traffic jams” where patients call from their cars before entering the clinic, which was one of the pandemic-related routines they had previously established to not exceed waiting room capacity. Taken together, they anticipated this policy change would require numerous changes to varied routines.

At the September 9th team meeting, JF announced that the Region put the clinic restart ‘on hold’, anticipating a fourth pandemic wave of infections. C clarified that it could happen with little notice if the potential surge of cases did not occur. JF suggested that, if schedules have capacity, they should take on new intakes in the shorter term, to avoid “getting too far behind” on the Region’s priorities for new intakes and larger panel sizes. Following up at the Sept 24th weekly update, C noted that clinic restart remains on hold but that patients are “demanding” to be seen in person for physicals, driving-related medical forms, and other issues. As a clinic, they decided that where possible, they would meet patient demand for in-person visits and re-initiate disease screening and immunizations.

The intake of new patients was made easier by another health policy change. Until Sept 2021, primary care providers’ time for the COVID-19 assessment clinic was taken from their time for new intakes. This resulted in fewer intakes, C mentioned at a weekly meeting (Sept 17), which is a primary care performance target tracked by the Region. On Oct 22, C added that the COVID-19 assessment clinic demands, in conjunction with the staff redeployment (and hence lost human resource capacity), also negatively impacted another key metric: time to third available appointment, related to advanced access. At that time, C shared that new funding has become available to the clinic from a COVID-19 cost centre, meaning that costs for the COVID-19 assessment clinic would no longer come from their primary care budget. The external policy funding change created more internal capacity for usual primary care functions.

In this example, several health policies – actual and anticipated – triggered varied self-organization processes. The priorities of external health policy makers had to be addressed in ways that were feasible within the clinic. Attempting to meet external policy demands revealed resource limits in terms of staffing and physical spaces, the challenges of infection control, and other external actors – in this case, patients and clinic funding – as influencing evolving, *tightly coupled* clinical and administrative routines.

### Self-organizing to address unintended consequences of earlier adaptations

As the prior example highlights, many pandemic-related changes that pre-existed our case study continued to have effects during the case study period. Prescription refill processes were another.

At the Oct 28/21 team meeting, C described how there seems to be a “HUGE” number of prescription refill faxes from pharmacies that are time consuming and “overwhelming” for administrative staff. The problem came to her attention via *feedback* from the primary care assistants and some primary care providers. She clarified that many patients have not been seen in-person for two years; “during COVID we have done a lot of prescription refills that we should not have done necessarily the same way without an appointment.” Through the team meeting conversation, they further *made sense* of the problem as one mainly affecting the part-time clinicians, where requests build up. The prescription refill backlog was an unintended consequence of the rapid switch to virtual care.

North Star clinic decided to start booking more patients for in-person appointments, to be assessed before refills prescribed (e.g., blood pressure measured, to support medication dosage adjustments if needed; urine testing of people with narcotics prescriptions). At the Nov 5th weekly meeting, C noted their recent change to clinical routines – booking more in-person appointments for patients – was not receiving “much pushback” so could continue. In summary, this problem was identified via *feedback* on difficulties, while limited negative *feedback* from patients helped them feel confident in the changes they made.

But changing the prescription refill routine created new problems for the lab, creating more demand for testing than existing supply. They needed to find a solution to the lab situation, given the lab results were necessary for primary care prescribing. They were still figuring out new routines to address this at the time we stopped data collection.

### Non-linear change dynamics: an example involving changing actors

At times, we observed how changing one actor in the actor-network can affect many clinical routines and require self-organization. In North Star, many routines depended on the stable co-leadership of the clinic. C and W, the administrative and medical leads, had worked together for over a decade, and their relationship had created a “seamlessness…almost like he and my roles had blended together in supporting the team” (C, Apr 5/22 report-back). But midway through the case study, W left the clinic, sparking a series of new processes of self-organization, some of which was unanticipated. Opioid deprescribing was one.

For context, North Star clinic had a long-standing interest in addiction medicine. Prior to the pandemic, some providers had secured licenses to prescribe suboxone, methadone, and the like (substantiated in document review). Pre-pandemic, changes to prescribing guidelines to address a growing overdose crisis had made physicians concerned about their licenses. And around the same time, Q, a pharmacist with an explicit interest in opioid stewardship, had started working there.

Then, a pandemic hit.

Opioid deprescribing came to our attention at a Sept 24/21 conversation with C, the administrative lead of the clinic. Several patients had contacted the clinic to request a new physician. C, then JF, the new medical lead, investigated to *make sense* of what was happening as per their pre-existing organizational routine for such requests. They found that most requests followed a new physician initiating a discussion about reducing opioid dosages with patients formerly on W’s panel. The leaders determined that these requests to change physician did not meet their policy threshold to do so. C added that the new physician was following newest guidelines endorsed by their licensure bodies regarding prescriptions of opioids, which recommends tapering of higher opioid doses for people taking the medication for chronic, non-cancer pain [42]. The problem, from prior clinical routines across the country which were more permissive of opioid prescribing, is “now we’ve got all these people pretty much addicted to pain medication” [C, Sept 24/21].

On Oct 1/21, C reported that the primary care providers met during the prior week to address “a fairly significant problem with opioid patients that have been left behind from when W left the practice”. She added that the prior routines were not working: “not only are the two providers that kind of took over W’s practice feeling uncomfortable, but the whole team is feeling a little bit more uncomfortable.” The new providers perceived pressure to reduce opioid use to align with their licensure College standards, and again expressed concern about losing their license if keeping the status quo. C further described how, due to COVID-19, telephone-based care and prescription refills by fax had resulted in many patients not completing the yearly recommended urine drug screen. This was an unintended consequence of prior self-organization done to adjust primary care services to pandemic conditions. The recent meeting reportedly allowed the group to *make sense* of the problem.

Routines did not immediately change. North Star opted to review, and if necessary, revise their ‘opioid contract’ (an electronic medical record template), re-introduce resources offered by Q (pharmacist), and for CP (physician) to propose that residents to consider a quality improvement project on opioid-related care. At the Oct 14/21 team meeting, JF described consensus among prescribing clinicians to prescribe opioids in ways consistent with then-current guideline and College of Family Physicians of Canada recommendations, with preparations underway to do so. The opioid contract review was ongoing, after having just received a copy of a recent licensure College document the day earlier from a physician at a different clinic. JF shared that the physician and nurse practitioners had decided to use discretion in terms of who to complete a contract with, given those conversations are sometimes “a bit confrontational”.

Yet this alone would not solve the problem of patients calling to request a new provider, which they anticipated would continue. L (senior primary care aide) and C confirmed an additional new routine was in place: when someone calls and asks to change providers, the primary care aide will instead book an appointment with the current one. If the patient still wishes to escalate, then they will be sent to C & JF, who will reiterate the process.

At the team meeting Oct 28/21, C gave the prescriber group a two-week time frame to discuss and finalize the new opioid contract. A new routine was not fully established by the time we exited the field.

The work observed to change clinical and administrative routines for opioid prescribing shows a range of influences on the outcome, but also an unexpected beginning: this trail of action starts with a long-term physician leaving the clinic. Patients calling to request a physician change is a type of *feedback*, which sparks a review of the situation by the clinic’s leadership. They clarified the problem as the result of implementation of new opioid prescribing practices in primary care, in relation to current external guidelines and fears of licensure loss. The prescribing primary care providers worked together to develop a new clinical routine and EMR resource, orienting to the new guideline more than patient’s concerns. But it is a routine they will enact at their discretion, taking into account the relationship impact such a change can have on a patient-provider relationship. The PCAs worked out a new administrative routine to manage calls from patients wishing to change providers. The changes to opioid-related care in the clinic is one example of unexpected clinical routine changes sparked from within, by new primary care clinicians joining the organization to replace W after his departure. While initiated from within, this change was also clearly influenced by external actors.

## Discussion

We aimed for our study to offer both methodological insight and a substantive case study of a primary care clinic as it navigated the second year of the pandemic. We address both in turn, in this discussion.

### Methodological insights

We sought to describe, in detail, the process that complexity scholars theorize as self-organization. While conceptually powerful, self-organization has eluded empirical description. For example, in Thompson and colleagues’ [22] review of applications of complexity theory in health services research, they found only seven studies where complexity theory guided the study from the outset and there was explicit attention to self-organization. Of these, four were qualitative studies that did not seek to study self-organization directly, instead relying on the concept to help interpret qualitative data collected for another purpose [22]. 

Starting from the understanding of self-organization as a process, we designed this case study to attempt to study directly the actions that took place. Applying methodological tenets from ANT, we focused our attention on action to alter organizational routines specifically. Doing so allowed us to make observable many complexity concepts. We address these strengths, then discuss limitations from our design choices and challenges.

First, we are able to demonstrate how each change is relational, [43] involving influence but not control, and occurred in an existing relational, sociomaterial context where the present is informed by the past [19]. Self-organization processes observed involved a conversational process of relating that complexity theorists call sense-making [44]. In complexity theory, there is no central controller; influence is enacted through relating with others, but not as a God or a designer [25]. While the administrator leader was a gatekeeper in terms of organizational routine changes – an “obligatory passage point”, to use Callon’s [33] term – decisions about changes came from conversations, often in meetings. Those conversations may happen prior to the administrative leader’s involvement, or at her initiation within a team meeting.

Second, this methodology gave us the tools to depict how humans and non-humans alike influence self-organization to create new routines. While some depictions of complexity theory emphasize human agents, actor-network theorists insist on taking non-human actors seriously as part of the relations that characterize actor-networks. A new virus, physical space, personal protective equipment, and technology were obvious non-human actors on self-organization; changing one created ripple effects, sometimes generating new problems. In the example of medication prescription refills, a change made in 2020 to refill prescriptions by fax became untenable by summer 2021. Addressing the problem required thinking through an approach that could fulfill ongoing public health orders for physical distancing in medical settings, which was limited by the physical spaces they had to work with.

Third, working with Braithewaite et al’s [10] definition, our methods were able to highlight the semi-autonomous aspect of self-organization, which some but not all definitions address. While the primary care clinic has some boundaries in terms of relationships, resources, and governance, co-located services and external bodies have an impact. For example, the capacity of the lab to manage demands for testing impacted primary care delivery. The primary care team can work with the lab to find a workable routine but could not themselves autonomously develop one.

Relatedly, we see a range of forms of feedback, both from within and externally, that spurred action in the clinic to re-develop clinical and organizational routines. Feedback may be formal, such as the performance report, or informal, such as “logjams” in processes or signals from patients, both of which were treated as feedback. Each spurred sensemaking processes among North Star members to try to understand the problem. And an absence of those signals – for example, patients not ‘pushing back’ on the request to attend in person – was a form of reassurance that the current routines were working.

Finally, using these methods, we can illuminate non-linearity. The departure of one physician, and hiring of new ones, led to an unanticipated series of actions to change opioid care practices. Other influences were folded in, including recent Canadian guidelines, and new physicians’ fears of licensure loss.

We also detected what organizational routine theorists call intersecting routines. This could take the form of tightly coupled routines, where changes to one trigger a cascade of additional changes. This was the case with the leadership change. But also, some self-organization processes intersect at the level of solutions; ‘clinic restart’ and prescription refills point to the same solution – more in-person care delivery.

In sum, we argue this methodological approach worked well to study self-organization, though with limits. Given the ongoing pandemic, we were limited to the use of virtual qualitative data collection methods. As well, we were unable to join and observe certain smaller group meetings, such as those among the primary care providers to talk about opioid deprescribing practices, or regular meetings among primary care aides. And some self-organization activities occur outside of formal meetings, though this could also be missed when in-person. Together, this meant not being able to directly observe some of the nuances in the actions, a limitation detected in member checking. While we did have access to team meetings – something argued as important [45] – at times, we were learning about the action shortly after it occurred, and filtered through the interpretation of the clinic member who updated us.

In addition, at time of design, we were advised that a written weekly tracking method filled in by multiple team members would likely work, if brief. The idea was for multiple members of the team to quickly fill in a weekly review. After difficulty initiating this in the participating clinic, we switched to a weekly call with the administrative leader, who we had already observed as the person through which passed all organizational routine changes (i.e., changes involving more than one person’s work). While these were rich and useful, we only have these updates for the latter half of our case and from one person’s perspective. If doing this type of work again, we will co-design and pilot the weekly update mechanism with the clinic before full initiation of the project. We anticipate structured weekly audiodiaries could work well for this purpose [46]. 

### Primary care practice, in the second year of the pandemic

Our case itself offers a substantive description of one primary care clinic weathering the pandemic’s second year. We are able to show how the effects of disequilibrium caused by the pandemic continued to unfold and how the clinic self-organized to navigate changes.

Much has been written about the rapid uptake of virtual care during the pandemic, but few studies to date give us a close-up view on primary care in its complexity during the pandemic. An exception is Russell and colleagues, [47] who explore the first ten months of pandemic-related changes in Australian general practice by recruiting a clinician researcher within each to prepare diary accounts. Their case study describes how organizational routines from pre-pandemic were far from useful under pandemic conditions; that is, the pandemic created disequilibrium.

In our study, we also capture the disequilibrium of the first year of the pandemic through retrospective interview accounts and document reviews. Our study picks up a later time point, showing how the changes of the first year continued to inform the routines and resources available in the second year of the pandemic. But we also partially were able to identify another source of disequilibrium: a long-term leader of the clinic leaving. Prior relationships that created a form of seamlessness of actions were no longer present; new relationships, new actor-networks needed to be developed. Together, these two studies help highlight how clinical and administrative routines can offer stability for a time, creating some order in ongoing disorder, however temporary – and that disequilibrium has different triggers.

At a broader level, Sturmberg and colleagues argue a core driver – a ‘key attractor’ in complexity terms – in primary care should be patient-centeredness and the patient-provider relationship [48]. Booth and colleagues’ longitudinal case of Australian general practice found competing drivers [7]; managers desired standardization, while clinicians oriented to creativity and autonomy to individualize. Our study brings others to the fore. First, ‘patient flow’ was repeatedly the reason for changing organizational routines. Problems with patient flow, whether anticipated or occurring, were the focus of much self-organization observed. Second, new family physicians initiated changes to opioid re-prescribing, which sparked broader self-organization across the clinic. Even though opioid deprescribing heightened conflict with patients, other drivers took precedence, including closer alignment with the then-current opioid prescribing guidelines, [42] and a commitment to within-clinic consistency to avoid additional problems. And our case study highlights an example of rapid, policy-required self-organization, where a core driver was infectious disease control. While required by policy, this concern was also shared by people within the organization. Core drivers are perhaps better thought of as plural, [12] aligning or misaligning in different ways in different situations over time.

## Conclusion

Many bemoan the slow rate of change in primary care. Yet, it is only at a distance that organizational routines look stable and an organization may look like it has inertia [39]. To study self-organization requires studying processes, that is, actions to re-establish a degree of equilibrium. As our case demonstrates, studying these change processes requires being close, using observational methods. By applying ANT methodological tenets to a case study involving observational, interview, and document data, we were able to illuminate how a primary care clinic is continually changing through this substantive case study of a Canadian primary care clinic in the second year of the pandemic. While the unique circumstances of the pandemic added urgency to some of the changes, our case study also highlights the sources of change are multiple in primary care. New government or insurer policies, leadership and other staff changes, clinical knowledge shifts, and more can all be an impetus for change. Like others before us, we argue that complexity dynamics better characterize primary care, offering an explanation why linear, ‘rational choice’, and planned change approaches fail [6, 7, 12]. 

We offer one methodological attempt to empirically study self-organization processes. However, we recognize that other approaches may also be fruitful. For example, orienting fully to organizational routines theory (rather than ANT) could be a meaningful foundation from which to develop methodologies to highlight self-organization. Future studies could build on the work of Tsoukas, who offers a theoretical conversation between complexity and organizational routines theories [19]. Among other possibilities, Tsoukas’ approach to organizations studies highlights the possibility of a different emphasis than our own – one on human experience, considering affective and motivational influences on organizational routines in addition to the “unfolding nature of organizational reality” [19]. ^p.148^ We anticipate a theoretical contrast between actor-network and organizational routines theory could also advance the methodological conversation.

### Supplementary Information

Supplementary Material 1.

## Data Availability

Not available.

## Abbreviations

ANT: Actor-network theory
EMR: Electronic medical record

## Footnotes

1 We use this term to refer to agents who encourage and/or work to initiate change.
