# Supplementary material for: Evolving through multiple, co-existing pressures to change: a case study of self-organization in primary care during the COVID-19 pandemic in Canada
Source: BMC Prim Care. 2024 Aug 5;25:285. doi: 10.1186/s12875-024-02520-3 (PMC11302186; doi:10.1186/s12875-024-02520-3)
Supplement: Supplementary file 1 — Supplementary Material 1. [file 12875_2024_2520_MOESM1_ESM.docx]

## **Appendix A: Data collection guides**

## **Interview guide**

Before we begin, I want to thank you for taking the time to speak with me today. There has been a lot going on, over a long period of time in many respects for your clinic and I appreciate your willingness to talk about the changes that have occurred.

Overview: As a reminder, the purpose of this project is to test methods of studying coexisting change pressures within primary care clinics. We are also trying to develop a robust case study of a clinic. Do you have any questions before we begin?

| **Questions** | Targeted Concepts |
| --- | --- |
| 1. Reflecting on the past year, can you remember times when the usual way of doing things were no longer working and changes were necessary. Note: not personally but change in terms of demands and clinic resources,  *Probes for each example:*         Please describe what led to this, internal and external.         What initiated this?         Was there something else contributing to this?    2. Of the changes made in the clinic, in the last year, what has been the most complex? What has made them so complex?           Probes to draw out iterations of changes:  – did it work on the first try? Did understanding the problem take time?  Is there EMR involved, new relationships need to be formed, new feedback systems, new ways of gathering information to check if it is working or what is best to do.    3. What things in the clinic were no longer working and needed to be changed. That is, in terms of demands and clinic resources, what changes were absolutely necessary?    Probes:   - things that necessitate action on an urgent basis – e.g., such as ‘you are becoming a vaccine clinic in two days time’; ‘next week we are redeploying 4 of 6 nurses’   If none, move to Q4. If yes, ask them to describe   - Probe what led to circumstance (?internal vs external-initiated change) - Can you recall other times when can you identify times when the usual routines in the clinic were no longer working and needed to be changed? (Probe multiple examples, as identified by the interviewee) | Disequilibrium    Drawing out actors relationships and conflicts.      Very far from equilibrium  I.e., the lack of fitness    (Is this lack of fitness or commitment to improvement) |
| 4.  a. How did your clinic respond to the need to makes changes to _________? (use language specific to the change mentioned by interviewee)  Probes:   - How did this work in the clinic? - What helped the clinic pull through this instability or instance of change?  Resources? Special skills? Other? - How did this influence staff interactions and/or relationships? - Did this change result in any unintended consequences? Please explain.   b. How was this change moved forward and by whom?  Probes;   - How were you involved in this change? How were others involved?   Was there feedback from others that helped shape how this evolved, or moved this change forward?  c. Can you recall other times when can you identify times when the usual routines in the clinic were no longer working and needed to be changed? (Probe multiple examples, as identified by the interviewee)    d. of these, which felt the hardest to face and why? [Probe for details] | Self-organization    Emergence  Sustainability,   feedback loops, interactions and relationships |
| 4 In the last year can you recall times when routines in the clinic seemed stable?  (Probes: draw from above) | Equilibrium |
| 5. Would you please tell me about an instance of expected (usual) versus unexpected (unusual) changes, and how you get through each instance? | Usual vs unusual change |

## 2. **Weekly structured update guide**

Opening (as needed): The purpose of the 15-minute weekly update is to see if this method adequately captures new change requests and identifies shifts related to ongoing change pressures. To recap, change requests should have the potential to change clinical care in ways that require new ways of working and/or people taking on new tasks. More specifically, change requests:

- Can be mandatory or optional.
- Demand communication and decision-making among multiple individuals who have different roles (i.e., more than one person needs to work on it to make it happen).
- Do not result in routine maintenance or temporary blips (i.e., not a thing to be worked around for a few days).

1. From the last (team meeting/weekly conversation), we know there are several changes underway. For example: (articulate list). Let’s start with ______. Please bring me up to speed on what has happened on _________?

Probes: If not already said:

(Looking forward) Whose hands is it in now? What comes next for that item?

(Looking back) What actions did folks here have to take to make it happen?

Is it going smoothly or are there complications to work through? If so, what are the complications?

[repeat as needed for each change underway]

2. What new change requests came in this week?

For each one:

a) From whom? What were they about?  What will happen next, if anything, for each new change? Can you give us a sense of number of new requests?

b) What is going to happen next for each of these new changes, if anything?

[repeat as needed for each new change request]

3. (As time allows) Is there something else that is influencing clinic functioning at the moment?
